# Randomized Phase II Trial of Sapanisertib ± TAK-117 vs. Everolimus in Patients With Advanced Renal Cell Carcinoma After VEGF-Targeted Therapy

**DOI:** 10.1093/oncolo/oyac192

**Published:** 2022-09-23

**Authors:** Toni K Choueiri, Camillo Porta, Cristina Suárez, John Hainsworth, Eric Voog, Ignacio Duran, James Reeves, Piotr Czaykowski, Daniel Castellano, Jingjing Chen, Farhad Sedarati, Thomas Powles

**Affiliations:** Dana-Farber Cancer Institute, Boston, MA, USA; University of Pavia and IRCCS San Matteo University Hospital Foundation, Pavia, Italy; Medical Oncology, Vall d’Hebron Institute of Oncology (VHIO), Hospital Universitari Vall d´Hebron, Vall d´Hebron Barcelona Hospital Campus, Barcelona, Spain; Sarah Cannon Research Institute, Nashville, TN, USA; Centre Jean Bernard/Clinique Victor Hugo, Institut Inter-régional de Cancérologie, Le Mans, France; Hospital Universitario Marqués de Valdecilla, IDIVAL, Santander, Cantabria, Spain; Florida Cancer Specialists/Sarah Cannon Research Institute, Fort Myers, FL, USA; CancerCare Manitoba, Winnipeg, Manitoba, Canada; i+12 Research Institute, Hospital Universitario 12 de Octubre, Madrid, Spain; Takeda Development Center Americas, Inc., Lexington, MA, USA; Takeda Development Center Americas, Inc., Lexington, MA, USA; Barts Cancer Institute, Royal Free NHS Trust, St. Bartholomew’s Hospital, London, UK

**Keywords:** everolimus, MTOR inhibitors, renal cell carcinoma, clinical trial, phase II

## Abstract

**Background:**

Sapanisertib, a dual mTORC1/2 inhibitor, may offer more complete inhibition of the PI3K/AKT/mTOR pathway than mTORC1 inhibitors, such as everolimus. This phase II study evaluated the efficacy and safety of single-agent sapanisertib and sapanisertib plus the PI3Kα inhibitor TAK-117, vs. everolimus in patients with advanced clear cell renal cell carcinoma (ccRCC) that had progressed on or after VEGF-targeted therapy.

**Materials and Methods:**

Patients with histologically confirmed, advanced ccRCC were randomized 1:1:1 to receive single-agent everolimus 10 mg once daily, single-agent sapanisertib 30 mg once weekly, or sapanisertib 4 mg plus TAK-117 200 mg, both once daily for 3 days/week, in 28-day cycles. The primary endpoint was progression-free survival (PFS).

**Results:**

Ninety-five patients were treated with everolimus or sapanisertib (*n* = 32 each), or sapanisertib plus TAK-117 (*n* = 31). There were no significant differences in PFS among the 3 groups or across any subgroups. Median PFS was 3.8 months with everolimus vs. 3.6 months with sapanisertib (HR, 1.33; 95% CI, 0.75-2.36), and 3.1 months with sapanisertib plus TAK-117 (HR, 1.37; 95% CI, 0.75-2.52). No significant differences in overall survival were seen among groups. Overall response rate was 16.7%, 0%, and 7.1%, respectively. Discontinuations due to treatment-emergent adverse events were 15.6%, 28.1%, and 29.0%.

**Conclusion:**

Sapanisertib with or without TAK-117 was less tolerable and did not improve efficacy vs. everolimus in patients with advanced ccRCC who had relapsed after or were refractory to VEGF-targeted therapies. Dual mTORC1/2 inhibition may not be an effective therapeutic approach for these patients.

## Teaser

This article evaluates the efficacy and safety of single-agent sapanisertib, and of sapanisertib plus the PI3Kα inhibitor TAK-117, versus everolimus in patients with advanced clear cell renal cell carcinoma that had progressed on or after VEGF-targeted therapy.

**Implications for Practice**

In this randomized phase II trial, treatment with the dual mTORC1/2 inhibitor sapanisertib, with or without the PI3Kα inhibitor TAK-117, appeared less tolerable and did not improve outcomes (progression-free survival or overall survival) compared with everolimus in patients with advanced or metastatic clear cell renal cell carcinoma that had progressed on or after VEGF-targeted therapy. Combined inhibition of mTORC1/2, with or without inhibition of additional targets in the PI3K/AKT pathway, remains an unproven therapeutic approach in these patients.

## Introduction

Advanced clear cell (cc) renal cell carcinoma (RCC) is a highly vascularized tumor type, which is largely chemoresistant^1,2^ and therefore commonly treated with antiangiogenic compounds, including tyrosine kinase inhibitors targeted to the vascular endothelial growth factor (VEGF) receptor and anti-VEGF antibodies.^3^ However, patients with ccRCC who initially respond to VEGF-targeted therapies will usually develop resistance to these agents.^4^

The rapalog (rapamycin analog) everolimus, a mammalian target of rapamycin complex 1 (mTORC1) inhibitor, is approved by the US Food and Drug Administration for the treatment of patients with advanced RCC after failure of treatment with the VEGF inhibitors sorafenib or sunitinib.^5^ However, as with VEGF-targeted therapies, resistance to rapalogs often develops after several months of treatment.^6^ This resistance is thought to be due to incomplete inhibition of mTORC1, or the abrogation of feedback inhibition leading to continued phosphoinositide 3-kinase (PI3K) signaling, and phosphorylation of AKT.^7-9^ Consequently, new treatment options that improve outcomes and prevent the development of resistance are needed for this patient population.^10-13^

Sapanisertib (TAK-228; MLN0128) is an investigational, orally bioavailable, and highly selective adenosine triphosphate-competitive inhibitor of both mTORC1 and mTORC2 that may improve upon the rapalog treatment response in metastatic ccRCC through more complete inhibition of mTORC1 signaling and blockade of mTORC2 substrates, such as AKT.^14-16^ In a phase I trial, single-agent sapanisertib demonstrated preliminary antitumor activity across different dosing schedules in patients with advanced RCC who had progressed on ≥1 anti-VEGF or mTORC1 inhibitor therapy (NCT01058707).^17^ One patient receiving sapanisertib 40 mg once weekly (QW) had a complete response (CR) and 7 patients had partial responses (PRs) (3 at 5 mg once daily [QD], one at 15 mg QW, one at 30 mg QW, and 2 at 40 mg QW).

TAK-117 (MLN1117) is an oral, potent, and highly selective small-molecule inhibitor of PI3Kα that blocks VEGF signaling and angiogenesis, while also inhibiting cellular phosphorylation and the activity of AKT *in vitro* and *in vivo*.^18,19^ The addition of a PI3Kα inhibitor to sapanisertib is theorized to prevent feedback reactivation of the PI3K/AKT/mTOR pathway and thus improve the response to sapanisertib through more complete and prolonged inhibition. In a first-in-human phase I clinical study (NCT01449370), TAK-117 demonstrated an acceptable safety profile and limited single-agent activity in patients with advanced solid malignancies.^19^ Further, preclinically in combination with sapanisertib, TAK-117 has demonstrated synergistic effects on the inhibition of tumor cell proliferation in bladder cancer cell lines, and on tumorigenesis and angiogenesis in xenograft models.^20^

Based on these preclinical and early clinical data, we conducted a phase II study to evaluate the efficacy and safety of single-agent sapanisertib and sapanisertib in combination with TAK-117 compared with everolimus in patients with advanced or metastatic ccRCC that had progressed on or after VEGF-targeted therapy; thus testing the hypothesis that dual mTORC1/2 inhibition, either with or without additional PI3Kα inhibition, will provide better efficacy than single-agent rapalog inhibition of mTORC1.

## Materials and Methods

### Study Design

This was a phase II, open-label, randomized, 3-arm study conducted at 35 centers in Europe (26 sites in the Czech Republic, France, Italy, Poland, Spain, and the UK) and North America (9 sites in Canada and the USA). The study was conducted according to the protocol, the ethical principles that have their origin in the Declaration of Helsinki, the International Conference on Harmonisation Guideline for Good Clinical Practice (E6), and all applicable laws and regulations. An institutional review board or independent ethics committee at each site reviewed and approved the study protocol and all amendments. Patients provided written informed consent. The trial was registered at clinicaltrials.gov (NCT02724020).

### Patients

Male or female patients aged ≥18 years with advanced or metastatic, histologically confirmed RCC with a clear-cell component, Karnofsky performance status ≥70%, life expectancy ≥3 months, and adequate organ function (including fasting serum glucose ≤130 mg/dL) were eligible. Patients must have received ≥1 prior line of VEGF-targeted therapy (but no more than 4 prior lines of systemic therapy in total) and have radiographic evidence of progressive disease (PD) according to Response Evaluation Criteria in Solid Tumors (RECIST) version 1.1,^21^ either on or within 6 months of stopping their most recent systemic therapy for RCC. Patients were excluded if they had central nervous system (CNS) metastases, clinically significant comorbidities that might compromise their participation in the study (such as uncontrolled pulmonary or cardiovascular disease, active CNS disease, or active infection), or if they had received prior treatment with agents that target PI3K, AKT, or mTOR.

### Study Treatment

Patients were stratified according to number of prior lines of therapy (1 vs. >1 prior line) and International Metastatic Renal Cell Carcinoma Database Consortium (IMDC) risk category (favorable vs. intermediate vs. poor) and randomized 1:1:1 to receive single-agent everolimus 10 mg QD, single-agent sapanisertib 30 mg QW (given on days 1, 8, 15, and 22), or sapanisertib 4 mg plus TAK-117 200 mg, both QD for 3 days per week (given on days 1-3, 8–10, 15-17, and 22-24), in 28-day treatment cycles. A centralized, interactive, voice- and/or web-based response system was used for randomization. Study treatment was given until disease progression, unacceptable toxicity, withdrawal of consent, or study closure.

### Study Endpoints

The primary endpoint was progression-free survival (PFS), defined as the time from the date of randomization to the date of first documentation of PD or death due to any cause, whichever occurred first. Evaluation of PD was based on investigator assessment of response per RECIST version 1.1. Secondary endpoints included overall survival (OS; defined as the time from the date of randomization to the date of death), best overall response rate (ORR; defined as CR plus PR, per RECIST version 1.1), clinical benefit rate (CBR; defined as CR plus PR plus stable disease), CBR at 16 weeks (CBR-16), and safety/tolerability. Health-related quality of life (HRQoL) endpoints were: change from baseline in functional and symptom subscale scores, and global health status/QoL score on the European Organisation for Research and Treatment of Cancer Quality of Life Questionnaire Core 30 (EORTC-QLQ-C30); and change from baseline in symptom scales on the Functional Assessment of Cancer Therapy-Kidney Symptom Index Disease-Related Symptoms (FKSI-DRS) questionnaire.

### Assessments

Radiographic tumor evaluations (contrast-enhanced computerized tomography scan of the chest, or magnetic resonance imaging with intravenous contrast of the abdomen and pelvis) were conducted by investigators at baseline (screening), every 3 cycles (on day 28) up to cycle 12, and then every 6 cycles (on day 28) thereafter (or per the investigators discretion), according to RECIST version 1.1.^21^ Treatment-emergent adverse events (TEAEs) were evaluated throughout the study and for 30 days after the last dose of study drug and graded according to the National Cancer Institute’s Common Terminology Criteria for Adverse Events (NCI-CTCAE) version 4.03. All patients treated with sapanisertib were given a glucometer to monitor their daily fasting blood glucose levels at home to assess hyperglycemia (defined as fasting blood glucose ≥150 mg/dL) as an on-target adverse event (AE). Other safety assessments included clinical laboratory parameters, vital signs, and 12-lead electrocardiograms. HRQoL was assessed on day 1 of each cycle and at the end of treatment using the EORTC QLQ-C30: a 30-item questionnaire incorporating 5 functional subscales (physical, role, emotional, cognitive, and social functioning), one global health status/QoL scale, 3 symptom subscales (fatigue, nausea/vomiting, and pain), and 6 single items (dyspnea, insomnia, appetite loss, constipation, diarrhea, and financial difficulties). The effect of disease-related symptoms on patients was evaluated at the same time points using FKSI-DRS, a validated, 9-item questionnaire derived from the 15-item FSKI-15 questionnaire.

### Statistical Analysis

Analyses of PFS, OS, and HRQoL were based on the full analysis set (all randomized patients), response analyses were based on the response-evaluable analysis set (patients who had received ≥1 dose of study drug with measurable disease at baseline and one post-baseline disease assessment), and safety analyses were based on the safety analysis set (all patients who had received ≥1 dose of study drug). The statistical analyses were conducted using SAS version 9.4. Missing data were not imputed for the efficacy analyses.

Time-to-event distributions were estimated using the Kaplan-Meier method. Hazard ratios (HRs) and 2-sided 95% confidence intervals (CIs) were estimated for the treatment comparisons (everolimus vs. sapanisertib, and everolimus vs. sapanisertib plus TAK-117) of PFS and OS using a stratified Cox regression model, with treatment arm and stratification factors as covariates. Treatment differences between arms were assessed using a 2-sided, stratified log-rank test. The primary hypothesis of PFS was tested at a 2-sided significance level of .15. A Cochran-Mantel-Haenszel (CMH) test adjusting for the stratification factors was used to compare ORR and CBR between the treatment arms. Changes from baseline in HRQoL scores were compared using a linear mixed model (random-intercept only model with unstructured covariance structure) with treatment arm, visit, interaction between treatment arm and visit, baseline score, and stratification factors as covariates.

Based on the assumptions that median PFS was 5 months with everolimus and that sapanisertib (either as a single agent or combined with TAK-117) could improve median PFS to 8 months (with a target HR of 0.625), a total of 95 PFS events were needed for each pairwise comparison to achieve approximately 80% power based on the 2-sided log-rank test at a significance level of .15 and a 10% dropout rate in each treatment arm. To achieve this number of PFS events, approximately 63 patients were required in each arm. Two interim analyses were planned; the first was performed after the first 30 patients in each arm had received ≥2 cycles of study medication, and a second interim analysis for futility was performed based on Bayesian posterior probability when 50% PFS events had occurred.

## Results

### Patients

**Figure 1.**
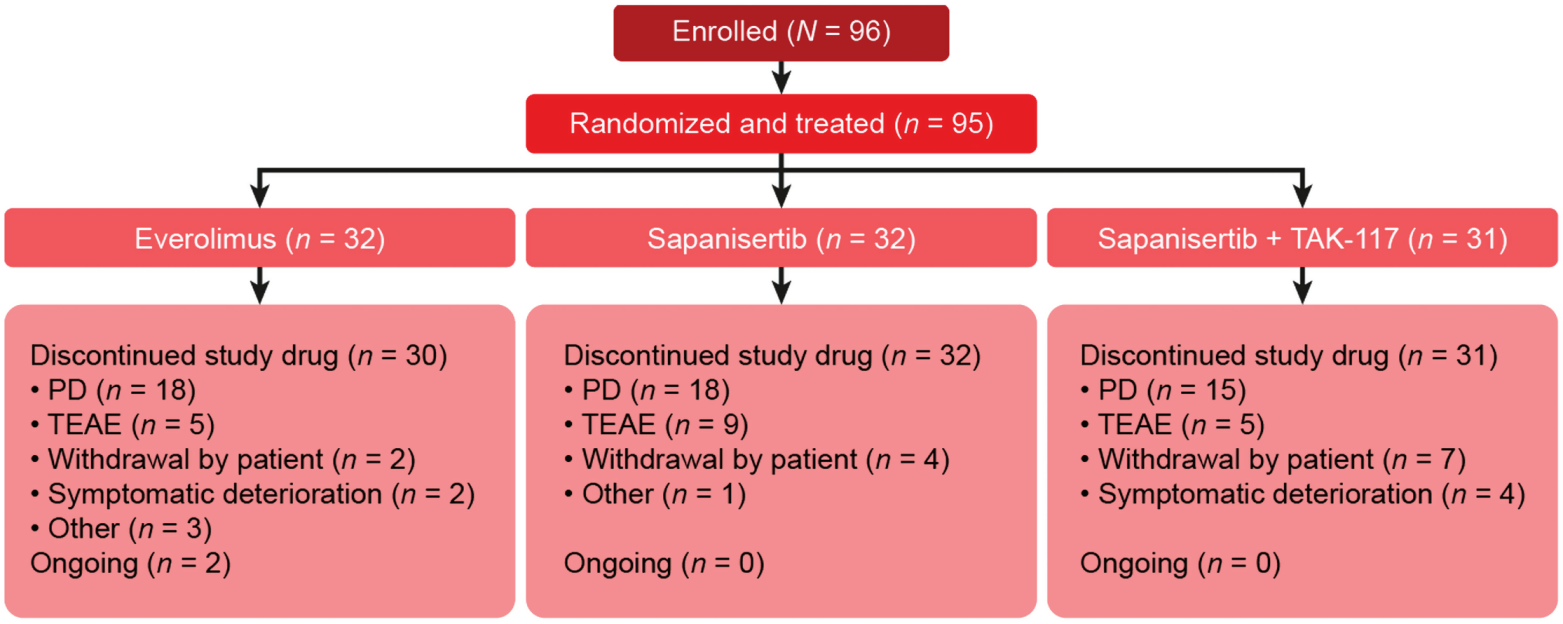
Patient disposition.

Abbreviations: PD, progressive disease; TEAE, treatment-emergent adverse event.

Enrollment for the study was stopped prematurely after the interim futility analysis (conducted after the first 68 patients had received ≥2 cycles of study medication) revealed higher discontinuations within the first 2 cycles and unfavorable efficacy for the sapanisertib-containing arms compared with the everolimus arm. At the time enrollment was stopped (March 6, 2020), 96 patients had been enrolled and randomized to receive everolimus (*n* = 32), single-agent sapanisertib (*n* = 32), or sapanisertib plus TAK-117 (*n* = 32; Fig. 1). One patient who was randomized to receive sapanisertib plus TAK-117 was not treated and was therefore excluded from the safety analysis set. Eighty-four patients were evaluable for response (30 in the everolimus arm, 26 in the sapanisertib arm, and 28 in the sapanisertib plus TAK-117 arm). At the time of data cut-off, 93 patients had discontinued treatment and 2 patients in the everolimus arm remained ongoing on study treatment. The most common reason for discontinuation of study treatment was PD (*n* = 51; 53.7%), followed by AEs (*n* = 19; 20.0%) and patient withdrawal (*n* = 13; 13.7%).

Patient baseline demographics and disease characteristics were generally well balanced among the 3 study arms with the exception of Karnofsky performance status scores and disease stage at study entry (Table 1). Across all arms, median age was 64.0 years (range, 35-81), median time from diagnosis to enrollment was 44.9 months (range, 3.8-419.8), 76.0% of patients had stage III or IV disease, 66.7% had received >1 prior line of therapy, and 78.1% had intermediate or poor IMDC risk. In the everolimus arm, there was a higher proportion of patients with a Karnofsky performance status score of 100 and a lower proportion with stage IV RCC, compared with the 2 sapanisertib arms (Table 1). Prior therapies are detailed in Supplementary Table S1. A similar proportion of patients in each arm had received prior immunotherapy (mainly nivolumab, 34.4%-40.6%).

**Table 1.** Baseline demographics and disease characteristics (full analysis set).

	Everolimus (*n* = 32)	Sapanisertib (*n* = 32)	Sapanisertib + TAK-117 (*n* = 32)	Total (*N* = 96)
Age, years				
Median (range)	66.0 (35-81)	61.0 (40-81)	66.0 (42-75)	64.0 (35-81)
≥65, *n* (%)	17 (53.1)	12 (37.5)	18 (56.3)	47 (49.0)
Male, *n* (%)	26 (81.3)	22 (68.8)	25 (78.1)	73 (76.0)
KPS, *n* (%)				
100	14 (43.8)	10 (31.3)	8 (25.0)	32 (33.3)
90	12 (37.5)	14 (43.8)	8 (25.0)	34 (35.4)
70-80	6 (18.8)	8 (25.0)	16 (50.0)	30 (31.2)
Disease stage at study entry^a^, *n* (%)				
II	4 (12.5)	4 (12.5)	1 (3.1)	9 (9.4)
III	9 (28.1)	7 (21.9)	8 (25.0)	24 (25.0)
IV	11 (34.4)	19 (59.4)	19 (59.4)	59 (51.0)
Unknown	8 (25.0)	2 (6.3)	4 (12.5)	14 (14.6)
IMDC risk category^b^, *n* (%)				
Favorable	6 (18.8)	8 (25.0)	7 (21.9)	21 (21.9)
Intermediate	22 (68.8)	20 (62.5)	19 (59.4)	61 (63.5)
Poor	4 (12.5)	4 (12.5)	6 (18.8)	14 (14.6)
Prior lines of therapy^b^, *n* (%)				
1	11 (34.4)	11 (34.4)	10 (31.3)	32 (33.3)
>1	21 (65.6)	21 (65.6)	22 (68.8)	64 (66.7)

Entry criteria required enrollment of patients with locally advanced or metastatic disease.

Prior lines of therapy (1 vs. >1) and IMDC risk category (favorable vs. intermediate vs. poor) were stratification factors.

Abbreviations: IMDC, International Metastatic RCC Database Consortium; KPS, Karnofsky performance status.

### Efficacy

**Figure 2.**
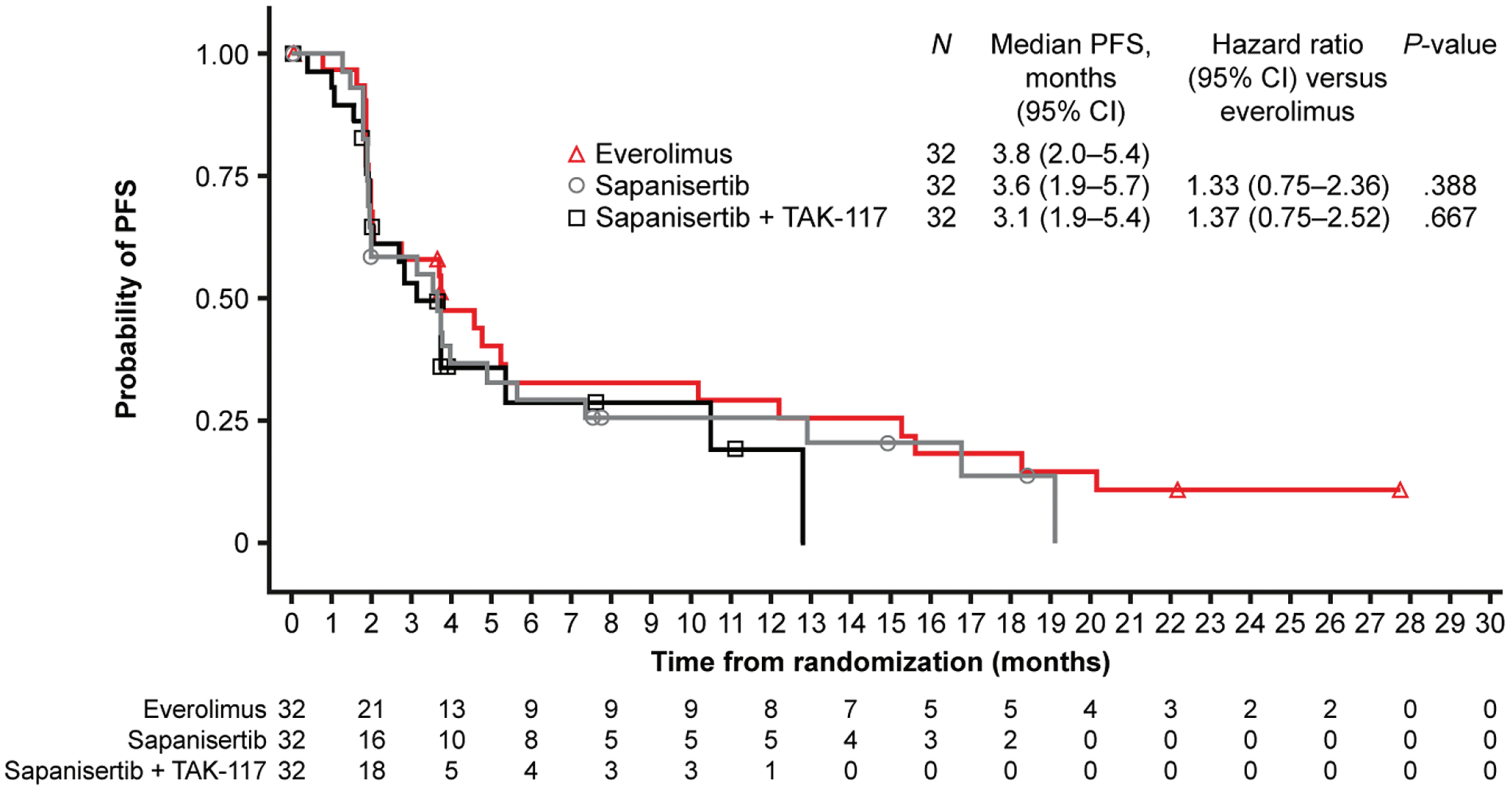
Progression-free survival (full analysis set). Abbreviations: CI, confidence interval; PFS, progression-free survival.

**Figure 3.**
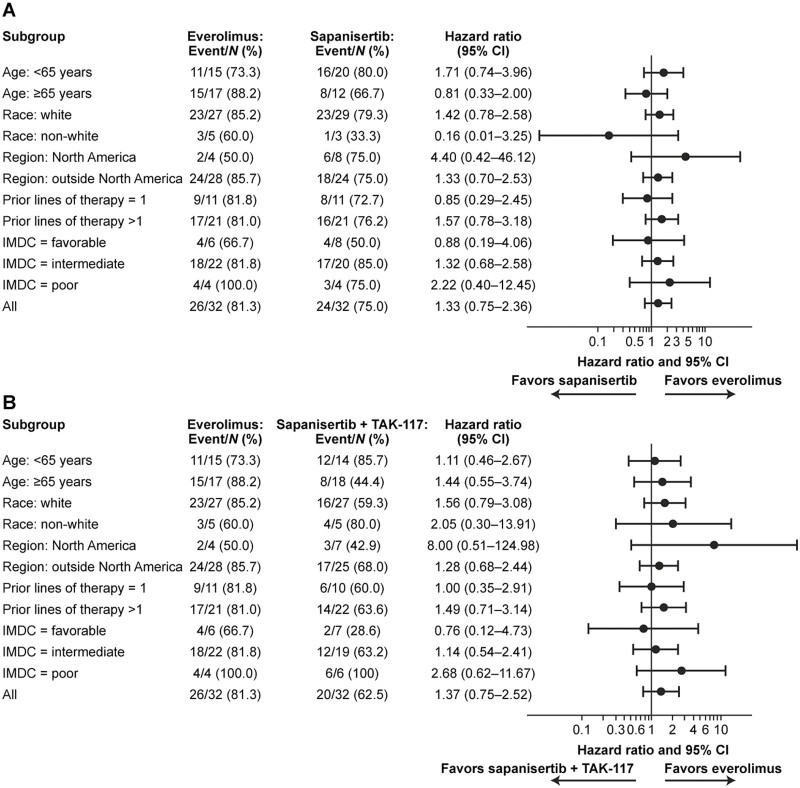
Forest plots of progression-free survival hazard ratios and 95% confidence intervals by patient subgroup, comparing: **(A)** everolimus vs. sapanisertib; and **(B)** everolimus vs. sapanisertib + TAK-117 (full analysis set). Abbreviations: CI, confidence interval; HR, hazard ratio; IMDC, International Metastatic RCC Database Consortium.

PFS was not significantly different between the everolimus treatment arm and either of the 2 sapanisertib arms (Fig. 2): median PFS was 3.8 months with everolimus compared with 3.6 months (HR, 1.33; 95% CI, 0.75-2.36; *P* = .388) with sapanisertib and 3.1 months (HR, 1.37; 95% CI, 0.75-2.52; *P* = .667) with sapanisertib plus TAK-117. Forest plots of PFS stratified by prespecified patient subgroups are shown for the comparisons of everolimus vs. single-agent sapanisertib in Fig. 3A, and everolimus vs. sapanisertib plus TAK-117 in Fig. 3B.

Median OS was 22.4 months in patients treated with everolimus vs. 16.2 months (HR, 1.76; 95% CI, 0.89-3.49; *P* = .212) in those who received single-agent sapanisertib and 18.1 months (HR, 1.51; 95% CI, 0.77-2.98; *P* = .546) in those treated with sapanisertib plus TAK-117; neither comparison was statistically significant (Supplementary Fig. S1). It should be noted that a lower proportion of patients in the everolimus arm (31.3%) received subsequent anticancer therapy compared with those in the single-agent sapanisertib (40.6%) and sapanisertib plus TAK-117 (71.0%) arms (Supplementary Table S2).

Best response is summarized by treatment arm in Table 2. In the everolimus arm, 5 of 30 evaluable patients achieved a PR for a confirmed ORR of 16.7%. No patients treated with single-agent sapanisertib achieved an objective response (odds ratio [OR] vs. everolimus not estimable). In the sapanisertib plus TAK-117 arm, 2 of 28 evaluable patients achieved a PR for a confirmed ORR of 7.1% (OR, 0.43; 95% CI, 0.08-2.29). Median duration of response was not estimable in any of the 3 arms. CBR was 66.7% in patients treated with everolimus, 61.5% in those treated with single-agent sapanisertib (OR, 0.84; 95% CI, 0.28-2.52), and 60.7% (OR, 0.85; 95% CI, 0.29–2.50) in those who received sapanisertib plus TAK-117. CBR-16 was 43.3%, 30.8% (OR, 0.52; 95% CI, 0.17-1.63), and 32.1% (OR, 0.63; 95% CI, 0.21-1.90), respectively.

**Table 2.** Summary of confirmed disease response by investigator assessment (response-evaluable analysis set).

Patients	Everolimus (*n* = 30)	Sapanisertib (*n* = 26)	Sapanisertib + TAK-117 (*n* = 28)
ORR, *n* (%)	5 (16.7)	0	2 (7.1)
CR	0	0	0
PR	5 (16.7)	0	2 (7.1)
SD, *n* (%)	15 (50.0)	16 (61.5)	15 (53.6)
SD ≥16 weeks	8 (26.7)	8 (30.8)	7 (25.0)
CBR, *n* (%)	20 (66.7)	16 (61.5)	17 (60.7)
CBR-16, *n* (%)	13 (43.3)	8 (30.8)	9 (32.1)

Abbreviations: CBR, clinical benefit rate (CR + PR + SD); CBR-16, CBR at 16 weeks; CR, complete response; ORR, overall response rate; PR, partial response; SD, stable disease.

### Safety and Tolerability

Patients received a median of 3.5 cycles of everolimus (range, 1-32), 3 cycles of single-agent sapanisertib (range, 1-20), and 3 cycles of sapanisertib plus TAK-117 (range, 1-17). Median relative dose intensity was 80.6% (range, 32.1-100%), 78.9% (range, 20.8%-100%), and 72.6% (range, 8.3%-101.2%), respectively.

The overall safety profile in each arm is summarized in Table 3, and the most common all-grade and grade ≥3 TEAEs are presented in Table 4. The most frequently reported TEAEs in patients treated with everolimus were asthenia (59.4%), decreased appetite (46.9%), diarrhea (40.6%), stomatitis (37.5%,) dyspnea (34.4%), and cough (34.4%). For patients treated with single-agent sapanisertib, the most commonly reported TEAEs were nausea (68.8%), vomiting (43.8%), asthenia (40.6%), and constipation and pruritus (both 37.5%). The most frequently reported TEAEs in patients treated with sapanisertib plus TAK-117 were nausea (54.8%), vomiting (41.9%), fatigue (38.7%), and diarrhea and decreased appetite (both 35.5%). Commonly reported grade ≥3 TEAEs by treatment arm were: pneumonia (9.4%), and fatigue, stomatitis, hyperglycemia, sepsis, and anemia (all 6.3%) in the everolimus arm; asthenia (12.5%), dyspnea (12.5%), and acute kidney injury and rash (both 9.4%) in the single-agent sapanisertib arm; and hyperglycemia and anemia (both 12.9%), and hypertension and abdominal pain (both 9.7%) in the combination arm. Incidence of serious AEs was 59.4%, 40.6%, and 48.4%, respectively.

**Table 3.** Tolerability and overall safety profile (safety analysis set).

	Everolimus (*n* = 32)	Sapanisertib (*n* = 32)	Sapanisertib + TAK-117 (*n* = 31)
Number of treatment cycles, median (range)	3.5 (1-32)	3.0 (1-20)	3.0 (1-17)
TEAEs, *n* (%)	32 (100)	30 (93.8)	31 (100)
Treatment-related TEAEs	31 (96.9)	28 (87.5)	27 (87.1)
Grade ≥3 TEAEs, *n* (%)	22 (68.8)	21 (65.6)	23 (74.2)
Treatment-related grade ≥3 TEAEs	14 (43.8)	11 (34.4)	14 (45.2)
SAEs, *n* (%)	19 (59.4)	13 (40.6)	15 (48.4)
Treatment-related SAEs	9 (28.1)	4 (12.5)	4 (12.9)
TEAEs leading to study discontinuation, *n* (%)	5 (15.6)	9 (28.1)	9 (29.0)
Treatment-related TEAEs leading to discontinuation	2 (6.3)	6 (18.8)	5 (16.1)
Treatment-related TEAEs leading to discontinuation in cycle 1 or 2	0	2 (6.3)	3 (9.7)
On-study deaths, *n* (%)	4 (12.5)	3 (9.4)	2 (6.5)

Abbreviations: SAE, serious adverse event; TEAE, treatment-emergent adverse event.

**Table 4.** Most frequently reported treatment-emergent adverse events (≥25% in any treatment arm) and grade ≥3 treatment-emergent adverse events (≥6% in any treatment arm) (safety analysis set).

Patients with ≥1 TEAE, *n* (%)	Everolimus (*n* = 32)		Sapanisertib (*n* = 32)		Sapanisertib + TAK-117 (*n* = 31)	
	All grade	Grade ≥3	All grade	Grade ≥3	All grade	Grade ≥3
Any TEAE	32 (100)	22 (68.8)	29 (90.6)	21 (65.6)	31 (100)	23 (74.2)
Asthenia	19 (59.4)	1 (3.1)	13 (40.6)	4 (12.5)	9 (29.0)	2 (6.5)
Decreased appetite	15 (46.9)	1 (3.1)	10 (31.3)	0	11 (35.5)	0
Diarrhea	13 (40.6)	1 (3.1)	8 (25.0)	0	11 (35.5)	0
Stomatitis	12 (37.5)	2 (6.3)	6 (18.8)	0	3 (9.7)	0
Dyspnea	11 (34.4)	1 (3.1)	11 (34.4)	4 (12.5)	5 (16.1)	0
Cough	11 (34.4)	0	10 (31.3)	0	3 (9.7)	0
Fatigue	10 (31.3)	2 (6.3)	6 (18.8)	2 (6.3)	12 (38.7)	2 (6.5)
Pyrexia	10 (31.3)	1 (3.1)	3 (9.4)	0	5 (16.1)	0
Constipation	9 (28.1)	0	12 (37.5)	1 (3.1)	5 (16.1)	1 (3.2)
Nausea	7 (21.9)	0	22 (68.8)	1 (3.1)	17 (54.8)	1 (3.2)
Vomiting	7 (21.9)	0	14 (43.8)	1 (3.1)	13 (41.9)	1 (3.2)
Hypertension	6 (18.8)	1 (3.1)	3 (9.4)	1 (3.1)	4 (12.9)	3 (9.7)
Pneumonia	5 (15.6)	3 (9.4)	1 (3.1)	0	0	0
Abdominal pain	5 (15.6)	0	5 (15.6)	1 (3.1)	6 (19.4)	3 (9.7)
Weight decreased	4 (12.5)	0	9 (28.1)	0	5 (16.1)	0
Hyperglycemia	4 (12.5)	2 (6.3)	4 (12.5)	0	8 (25.8)	4 (12.9)
Anemia	4 (12.5)	2 (6.3)	6 (18.8)	1 (3.1)	5 (16.1)	4 (12.9)
Pruritus	3 (9.4)	0	12 (37.5)	1 (3.1)	6 (19.4)	0
Rash	3 (9.4)	0	3 (9.4)	3 (9.4)	5 (16.1)	0
Sepsis	2 (6.3)	2 (6.3)	1 (3.1)	1 (3.1)	0	0
Acute kidney injury	0	0	3 (9.4)	3 (9.4)	1 (3.2)	0
Alanine aminotransferase increase	0	0	1 (3.1)	1 (3.1)	4 (12.9)	2 (6.5)
Ascites	0	0	0	0	0	2 (6.5)
Blood creatinine increase	2 (6.3)	1 (3.1)	3 (9.4)	2 (6.3)	2 (6.5)	0
Metastatic renal cell carcinoma	0	0	2 (6.3)	2 (6.3)	0	0

Abbreviation: TEAE, treatment-emergent adverse event

TEAEs leading to discontinuation from the study were observed in 15.6% of patients in the everolimus arm, 28.1% of those in the sapanisertib arm, and 29.0% of patients in the sapanisertib plus TAK-117 arm (Table 3). Treatment-related TEAEs leading to discontinuation were seen in 6.3% (0% in cycle 1 or 2), 18.8% (6.3% in cycle 1 or 2), and 16.1% (9.7% in cycle 1 or 2) of patients, respectively. Two patients treated with everolimus discontinued due to treatment-related pneumonia and sepsis (*n* = 1 each). Six patients who received single-agent sapanisertib discontinued due to treatment-related acute kidney injury, asthenia, fatigue/muscular weakness, nausea, pneumonitis, and septic shock (*n* = 1 each). Five patients treated with sapanisertib plus TAK-117 discontinued due to treatment-related alanine aminotransferase increase, fatigue, general physical health deterioration, systemic inflammatory response syndrome, and transaminases increase (*n* = 1 each).

Nine patients died while on study: 4 (12.5%) in the everolimus arm, 3 (9.4%) in the sapanisertib arm, and 2 (6.5%) in the sapanisertib plus TAK-117 arm. Of these, four deaths were considered to be treatment related: 2 in the everolimus arm (due to sepsis and pneumonia, respectively), one in the sapanisertib arm (due to septic shock) and one in the sapanisertib plus TAK-117 arm (due to general physical health deterioration).

### Health-Related Quality of Life

There was a high level of compliance across all 3 arms for completion of the EORTC QLQ-C30 (91.3%-95.9%) and FKSI-DRS (88.2%-95.2%) questionnaires. All HRQoL analyses were limited to the first 6 cycles as none of the 3 treatment arms included >10 patients after cycle 6.

In all 3 treatment arms, patients showed a trend for deterioration from baseline in HRQoL and disease-related symptoms, as indicated by the mean change from baseline in EORTC QLQ-C30 (Supplementary Fig. S2A) and FKSI-DRS (Supplementary Fig. S2B) summary scores, and EORTC QLQ-C30 global health status/QoL score (Supplementary Fig. S2C). Differences between the everolimus arm and the 2 sapanisertib arms in the mean changes from baseline in these scores were not consistently, significantly different (*P* <.05) in the first 4 cycles. However, compared with patients in the everolimus arm, patients in the single-agent sapanisertib arm showed greater deterioration from baseline in the EORTC QLQ-C30 subscales of emotional functioning (Supplementary Fig. S2D), cognitive functioning (Supplementary Fig. S2E), and role functioning (Supplementary Fig. S2F), and in the symptom subscales of nausea/vomiting (Supplementary Fig. S2G) and insomnia (Supplementary Fig. S2H); differences in mean changes from baseline were statistically significant for all these scores at cycle 3 (*P* <.05).

## Discussion

In this phase II study, patients with advanced or metastatic ccRCC were randomly assigned to receive the approved mTORC1 inhibitor everolimus (as control), the investigational dual mTORC1/2 inhibitor sapanisertib, or sapanisertib combined with the experimental PI3Kα inhibitor TAK-117. Enrollment was closed early after a per-protocol assessment of the first 68 patients who had received ≥2 cycles of study drug indicated higher discontinuations within the first 2 cycles and no evidence of improved efficacy for the sapanisertib-containing arms compared with everolimus. Consequently, the study was no longer powered to assess a difference in the primary endpoint of PFS. At the time of the analysis, there was no improvement in the primary endpoint of PFS among patients treated with sapanisertib or sapanisertib plus TAK-117 compared with everolimus, either in the overall study population or in any of the prespecified patient subgroups. A lack of clinical benefit with either sapanisertib or sapanisertib plus TAK-117 compared with everolimus was also consistently observed across the secondary endpoints of OS, ORR, CBR, and CBR-16. It should be mentioned, however, that these results could have been affected by an imbalance in baseline characteristics among the treatment arms, with everolimus-treated patients tending to have a slightly better performance status and less advanced disease.

It was thought that the use of sapanisertib as a dual mTORC1/2 inhibitor may improve upon the efficacy outcomes that can be achieved with everolimus, which only partially inhibits the mTOR pathway via mTORC1 but not mTORC2.^22^ It was also thought that the addition of TAK-117 to sapanisertib would provide more robust mTOR inhibition compared with single-agent sapanisertib through upstream inhibition of mTORC1/2 signaling and potentially mitigating feedback activation of AKT, which facilitates resistance to rapalogs.^23^ The consistent lack of clinical benefit in this study in patients treated with sapanisertib, beyond that achieved with everolimus, suggests that dual mTORC1/2 inhibition, either alone or with PI3Kα inhibition, may not be an effective clinical approach for the treatment of patients with advanced or metastatic ccRCC who have relapsed or are refractory to VEGF-targeted therapies. It has been suggested that mutations in close upstream regulators of *mTOR* and loss of phosphatase and tensin homolog (PTEN) expression may be more reliable predictors of response to rapalogs than histological subtype.^24^ Thus, the selection of patients with ccRCC may not have been ideal for treatment with combination rapalog/mTOR inhibitors. Due to the heterogeneity of mechanisms involved in PI3K/AKT/mTOR activation in ccRCC, a more targeted approach is needed to predict patients benefiting from rapalogs. However, reliable and robust biomarkers are not yet available for ccRCC.^24^ Biomarkers predictive of response were not assessed in this study and thus, the effect of PTEN expression on response to sapanisertib in these patients is unknown.

It is notable that 2 other small molecule dual inhibitors of mTORC1/2, AZD2014 and apitolisib, also failed to improve upon the efficacy of everolimus in randomized phase II trials in patients with VEGF-refractory metastatic RCC.^25^ For these 2 molecules, PFS was significantly worse compared with everolimus; an observation that was not seen with sapanisertib in the present study, where outcomes were similar across study arms. No apparent relationship was identified between biomarker mutations, including *PTEN* mutations, and best response to apitolisib.^25^ Biomarkers were not assessed in the trial of AZD2014.^26^ The phase II trial of apitolisib and a phase Ib study of another similar, small molecule inhibitor BEZ235, both of which target PI3K along with mTORC1 and mTORC2, also indicate that targeting PI3K/AKT/mTOR signaling can result in significant toxicity, thus limiting any potentially achievable clinical benefits.^25,27^ The lack of benefit with dual mTORC1/2 inhibition could also suggest a peripheral role of mTORC2 in the PI3K/AKT/mTOR pathway in advanced RCC and/or emergence of resistance to mTORC1 inhibition through activation of parallel, compensatory signaling pathways that promote tumor cell survival.^28,29^

The safety profile of sapanisertib was similar with or without the addition of TAK-117; however, both sapanisertib treatment arms appeared less tolerable than the everolimus arm, as reflected by the higher proportion of discontinuations due to treatment-related TEAEs, particularly during the first 2 cycles of treatment. As stated previously, studies of other mTORC1/2 inhibitors in advanced RCC have also reported issues with tolerability.^25,27^ The most frequent TEAEs reported with sapanisertib or sapanisertib plus TAK-117 were nonetheless consistent with previous studies of single-agent sapanisertib^16,17,30^ and were generally in line with the pharmacodynamic mechanism of mTOR and PI3K inhibitors. No new safety signals were reported compared with previous studies. Patients in all 3 arms showed a deterioration in their HRQoL and disease-related symptoms based on EORTC QLQ-C30 and FKSI-DRS assessments, but this was particularly pronounced in patients receiving single-agent QW sapanisertib. The higher rates of nausea and vomiting TEAEs observed with single-agent sapanisertib were reflected in higher (worse) scores in the EORTC QLQ-C30 symptom subscale of nausea/vomiting, particularly at cycles 3.

## Conclusion

In this phase II study, treatment with sapanisertib with or without TAK-117 appeared less tolerable and did not demonstrate improved efficacy vs. everolimus in patients with advanced or metastatic ccRCC. Combined inhibition of mTORC1/2, with or without inhibition of additional targets in the PI3K/AKT pathway, remains an unproven therapeutic approach for these patients.

## Supplementary Material

oyac192_suppl_Supplementary_Figure_S1

oyac192_suppl_Supplementary_Figure_S2

oyac192_suppl_Supplementary_Table_S1

oyac192_suppl_Supplementary_Table_S2

## Data Availability

Requests for de-identified datasets for the results reported in this publication will be made available to qualified researchers following submission of a methodologically sound proposal. Data will be made available for such requests following online publication of this article and for 1 year thereafter in compliance with applicable privacy laws, data protection, and requirements for consent and anonymization. Calithera does not share identified participant data or a data dictionary.

## Notes

ClinicalTrials.gov Identifier: NCT02724020
