# Supplementary material for: Randomized Phase II Trial of Sapanisertib ± TAK-117 vs. Everolimus in Patients With Advanced Renal Cell Carcinoma After VEGF-Targeted Therapy
Source: Oncologist. 2022 Sep 23;27(12):1048–57. doi: 10.1093/oncolo/oyac192 (PMC9732228; doi:10.1093/oncolo/oyac192)
Supplement: oyac192_suppl_Supplementary_Figure_S1 [file oyac192_suppl_supplementary_figure_s1.docx]

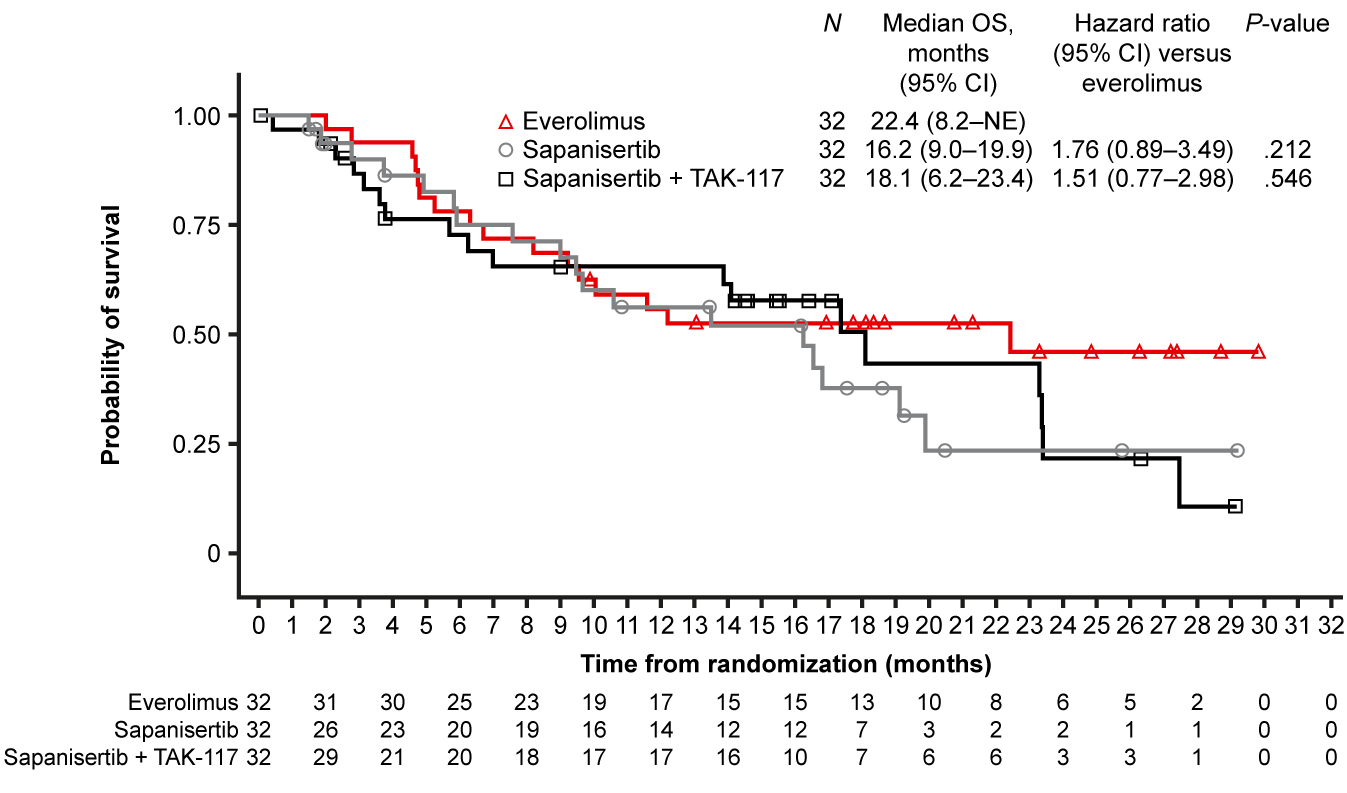


**Supplemental Figure 1.** Overall survival (full analysis set).

Abbreviations: CI, confidence interval; NE, non-estimable; OS, overall survival.
