# Supplementary material for: Randomized Phase II Trial of Sapanisertib ± TAK-117 vs. Everolimus in Patients With Advanced Renal Cell Carcinoma After VEGF-Targeted Therapy
Source: Oncologist. 2022 Sep 23;27(12):1048–57. doi: 10.1093/oncolo/oyac192 (PMC9732228; doi:10.1093/oncolo/oyac192)
Supplement: oyac192_suppl_Supplementary_Figure_S2 [file oyac192_suppl_supplementary_figure_s2.docx]

**
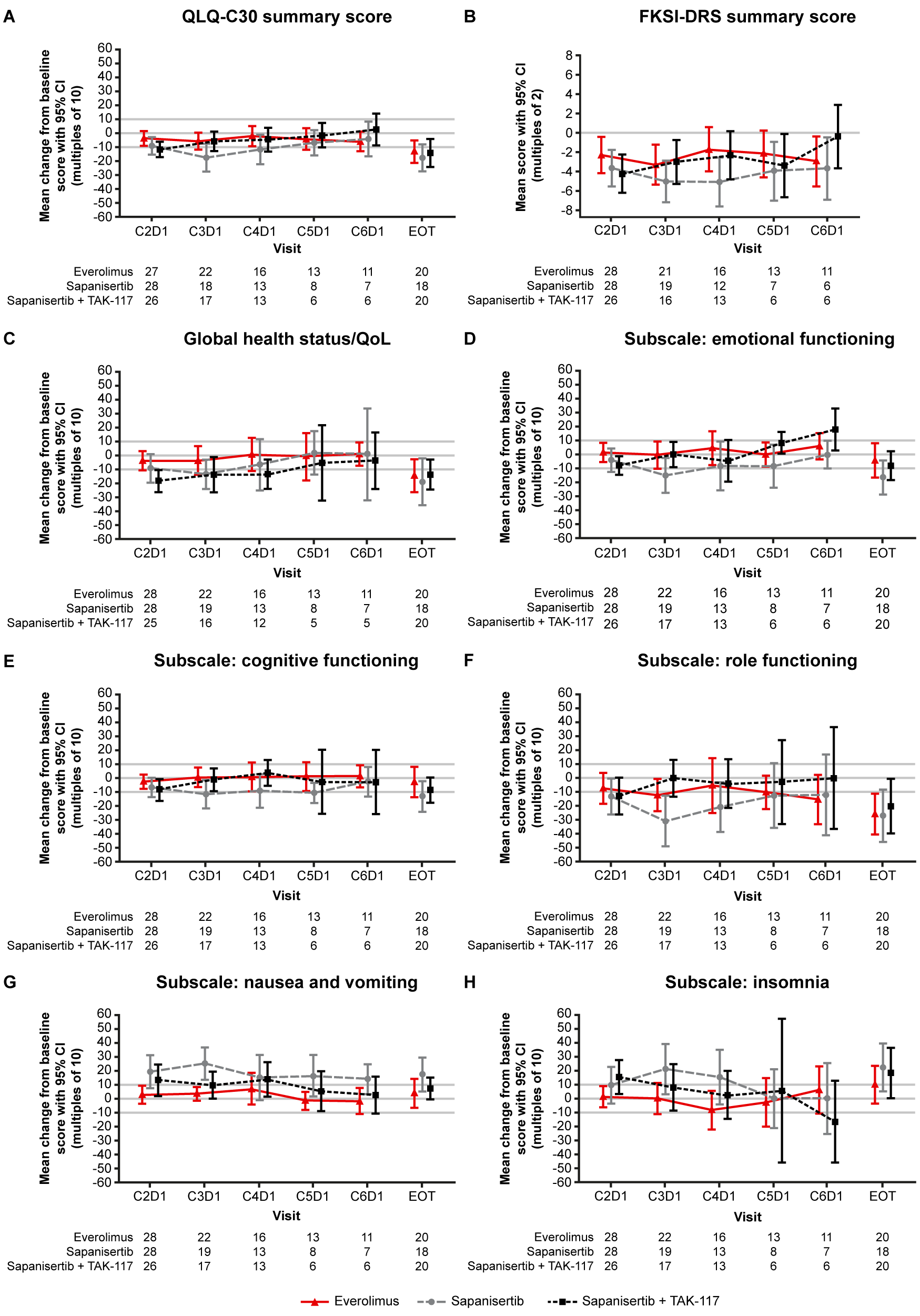
Supplemental Figure 2.** Mean change from baseline in health-related quality of life and disease-related symptoms: **(A)** EORTC QLQ-C30 summary score; **(B)** FKSI-DRS summary score; **(C)** EORTC QLQ-C30 global health status/QoL score; **(D)** EORTC QLQ-C30 emotional functioning subscale score; **(E)** EORTC QLQ-C30 cognitive functioning subscale score; **(F)** EORTC QLQ-C30 role functioning subscale score; **(G)** EORTC QLQ-C30 nausea and vomiting symptom subscale score; and **(H)** EORTC QLQ-C30 insomnia symptom subscale score.

Abbreviations: C, cycle; CI, confidence interval; D, day; EORTC QLQ-C30, European Organisation for Research and Treatment of Cancer Quality-of-life Questionnaire Core 30; EOT, end of treatment; FKSI-DRS, Functional Assessment of Cancer Therapy-Kidney Symptom Index Disease-Related Symptoms; QoL, quality of life.
