# Supplementary material for: Randomized Phase II Trial of Sapanisertib ± TAK-117 vs. Everolimus in Patients With Advanced Renal Cell Carcinoma After VEGF-Targeted Therapy
Source: Oncologist. 2022 Sep 23;27(12):1048–57. doi: 10.1093/oncolo/oyac192 (PMC9732228; doi:10.1093/oncolo/oyac192)
Supplement: oyac192_suppl_Supplementary_Table_S1 [file oyac192_suppl_supplementary_table_s1.docx]

**SUPPLEMENTARY MATERIALS**

**Supplemental Table 1.** Prior therapies (safety analysis set).

|  | **Everolimus** | **Sapanisertib** | **Sapanisertib**  **+ TAK-117** | **Total** |
| --- | --- | --- | --- | --- |
|  | **(*n* = 32)** | **(*n* = 32)** | **(*n* = 31)** | **(*N* = 95)** |
| Prior lines of therapy, *n* (%)^a^ |  |  |  |  |
| 1 | 10 (31.3) | 11 (34.4) | 8 (25.8) | 29 (30.5) |
| 2 | 12 (37.5) | 7 (21.9) | 8 (25.8) | 27 (28.4) |
| 3 | 9 (28.1) | 9 (28.1) | 11 (35.5) | 29 (30.5) |
| 4 | 1 (3.1) | 4 (12.5) | 3 (9.7) | 8 (8.4) |
| 5 | 0 | 1 (3.1) | 1 (3.2) | 2 (2.1) |
| Prior lines of VEGF-targeted therapy, *n* (%) |  |  |  |  |
| 1 | 18 (56.3) | 15 (46.9) | 13 (41.9) | 46 (48.4) |
| 2 | 12 (37.5) | 11 (34.4) | 15 (48.4) | 38 (40.0) |
| 3 | 2 (6.3) | 6 (18.8) | 3 (9.7) | 11 (11.6) |
| Type of VEGF-targeted therapy, *n* (%) |  |  |  |  |
| Sunitinib | 22 (68.8) | 22 (68.8) | 25 (80.6) | 69 (72.6) |
| Pazopanib | 10 (31.3) | 10 (31.3) | 12 (38.7) | 32 (33.7) |
| Axitinib | 9 (28.1) | 13 (40.6) | 6 (19.4) | 28 (29.5) |
| Cabozantinib | 4 (12.5) | 4 (12.5) | 3 (9.7) | 11 (11.6) |
| Bevacizumab | 2 (6.3) | 2 (6.3) | 4 (12.9) | 8 (8.4) |
| Sorafenib | 1 (3.1) | 3 (9.4) | 1 (3.2) | 5 (5.3) |
| Tivozanib | 0 | 1 (3.1) | 1 (3.2) | 2 (2.1) |
| Other types of prior therapy, *n* (%) |  |  |  |  |
| Nivolumab | 11 (34.4) | 13 (40.6) | 12 (38.7) | 36 (37.9) |
| Atezolizumab | 2 (6.3) | 2 (6.3) | 2 (6.5) | 6 (6.3) |
| Durvalumab | 0 | 1 (3.1) | 1 (3.2) | 2 (2.1) |
| Savolitinib | 0 | 1 (3.1) | 1 (3.2) | 2 (2.1) |
| Other | 2 (6.3) | 3 (9.4) | 4 (12.9) | 9 (9.5) |

^a^Based on data from electronic case report forms (these differ from the data shown in Table 1, which are based on data from the interactive response system).

Abbreviation: VEGF, vascular endothelial growth factor.
