# Supplementary material for: Randomized Phase II Trial of Sapanisertib ± TAK-117 vs. Everolimus in Patients With Advanced Renal Cell Carcinoma After VEGF-Targeted Therapy
Source: Oncologist. 2022 Sep 23;27(12):1048–57. doi: 10.1093/oncolo/oyac192 (PMC9732228; doi:10.1093/oncolo/oyac192)
Supplement: oyac192_suppl_Supplementary_Table_S2 [file oyac192_suppl_supplementary_table_s2.docx]

**Supplemental Table 2.** Subsequent anticancer therapies (safety analysis set).

|  | **Everolimus** | **Sapanisertib** | **Sapanisertib**  **+ TAK-117** | **Total** |
| --- | --- | --- | --- | --- |
|  | **(*n* = 32)** | **(*n* = 32)** | **(*n* = 31)** | **(*N* = 95)** |
| Patients with any subsequent anticancer therapy, *n* (%) | 10 (31.3) | 13 (40.6) | 22 (71.0) | 45 (47.4) |
| Type of anticancer therapy, *n* (%) |  |  |  |  |
| Cabozantinib | 2 (20.0) | 7 (53.8) | 10 (45.5) | 19 (42.2) |
| Nivolumab | 4 (40.0) | 5 (38.5) | 10 (45.5) | 19 (42.2) |
| Axitinib | 3 (30.0) | 1 (7.7) | 2 (9.1) | 6 (13.3) |
| Everolimus | 0 | 2 (15.4) | 3 (13.6) | 5 (11.1) |
| Sorafenib | 1 (10.0) | 1 (7.7) | 1 (4.5) | 3 (6.7) |
| Lenvatinib | 1 (10.0) | 0 | 1 (4.5) | 2 (4.4) |
| Pazopanib | 0 | 1 (7.7) | 1 (4.5) | 2 (4.4) |
| Tivozanib | 1 (10.0) | 1 (7.7) | 0 | 2 (4.4) |
| Bevacizumab | 0 | 0 | 1 (4.5) | 1 (2.2) |
| BO-112 | 1 (10.0) | 0 | 0 | 1 (2.2) |
| Durvalumab | 1 (10.0) | 0 | 0 | 1 (2.2) |
| Radiotherapy L1-2 | 0 | 1 (7.7) | 0 | 1 (2.2) |
| Temsirolimus | 0 | 1 (7.7) | 0 | 1 (2.2) |
